# Society Proceedings

**Published:** 1903-01

**Authors:** 


### SOCIETY PROCEEDINGS.

### PASSAIC COUNTY VETERINARY MEDICAL ASSOCIATION.

The regular monthly meeting of the Passaic County Veterinary Medical Association was held at 169 Paterson street, Paterson, N. J., on Tuesday evening, December 2, 1902, with Dr. William Herbert Lowe, President, in the chair, and Dr. William J. Fredericks acting as Secretary.

On roll-call the following members answered to their names: Drs. Anthony P. Lubach, Passaic; J. Payne Lowe, Passaic; John H. De Graw, Paterson; David Machan, Paterson; T. J. Cooper, Paterson; William H. H. Doty, Paterson; M. A. Pierce, Paterson; William J. Fredericks, Delawanna, and William Herbert Lowe, Paterson.

The minutes of November 4 were read and approved on motion of Dr. J. Payne Lowe, seconded by Dr. Cooper.

The next order of business being "unfinished business of the previous meeting," the President reported that the certificates of membership had been printed as ordered, at an expense of $5, and that a number of members already had their certificates, each paying fifty cents for the same, which from our nineteen members would bring into the treasury $9.50, so that the Association was actually making $4.50 by the transaction. On motion the report was received and ordered entered upon the minutes.

Dr. Cooper was allowed to withdraw his "blacklist" proposition by unanimous consent.

Treasurer M. A. Pierce made the following report:

*Receipts.*

| | | |
|---|---|---|
| September 16th. Received from Dr. A. Machan, Secretary . . . . . . . . | $16.00 | |
| October 31st. Received from Dr. A. Machan, Secretary . . . . . . . . | 10.00 | |
| | | $26.00 |

*Disbursements.*

| | | |
|---|---|---|
| Paid Guardian P. & P. Co.'s bill for 200 letter-heads, 200 envelopes, 100 postal cards, and 200 schedule of fee cards . . . . | $9.75 | |
| Paid the same company's bill for 250 copies of by-laws . . . . . . . . . | 9.75 | |
| Paid the same company's bill for fifty certificates . | 5.00 | |
| | | 24.50 |
| Balance on hand . . . . . . . . . . | | $1.50 |

It was regularly moved, seconded, and carried that the Treasurer's report be received and spread in full upon the minutes.

The President then paid over to the Treasurer $11.50, which had been paid to him since the last meeting for certificates and dues.

President Lowe read part of a letter from Dr. W. Horace Hoskins of Philadelphia, managing editor of THE JOURNAL OF COMPARATIVE MEDICINE AND VETERINARY ARCHIVES, in which the editor said that he noted "the continued interest of your members in the work of the profession, and trusts that it will continue with added power and influence."

The President reported that he had received a letter from Dr. Roscoe R. Bell, of Brooklyn, N. Y., editor of *The American Veterinary Review* advising him that the December *Review* would contain the full reports of the last three meetings of the Passaic County Veterinary Medical Association.

The President then made the following announcements: Next meeting of the Passaic County Veterinary Medical Association, Tuesday evening, January 6, 1903.

Annual meeting of the Veterinary Medical Association of New Jersey, at the Trenton House, Trenton, N. J., Thursday, January 8, 1903. One of the most interesting and instructive features of the programme to practitioners would be a lantern-slide exhibition and address on "The Etiology and Prevention of Infectious Diseases of Animals," by Prof. Veranus A. Moore, of the New York State Veterinary College, at Cornell University, an eminent authority on comparative pathology and bacteriology.

Meeting of the State Board of Veterinary Medical Examiners at the State House, Trenton, N. J., January 9 and 10, 1903, for the examination of candidates for license to practice veterinary medicine, surgery, and dentistry in the State of New Jersey.

The members resolved to attend the State meeting at Trenton on January 8 in a body.

The Association then listened with much interest to the reading of a paper on the "Examination of Horses as to Soundness," by Dr David Machan. A profitable discussion followed on several aspects of this important subject to horsemen and veterinarians. The Association gave Dr. Machan a vote of thanks.

The President appointed Dr. Fredericks essayist for the January meeting.

On motion, the meeting adjourned at 10.45 P.M.

WILLIAM J. FREDERICKS,
Secretary *pro tem.*

---

### KEYSTONE VETERINARY MEDICAL ASSOCIATION.

THE regular monthly meeting of the Keystone Veterinary Medical Association was held at the usual place, December 9, 1902. President Kooker called the meeting to order, and in the absence of the Secretary, Dr. W. Horace Hoskins was appointed Secretary *pro tem.*

The members of the profession present were Drs. Carter, Conard, Cox, M. W. Drake, Hoskins, Kooker, Lintz, Marshall, Mecray, Thomas B. Rayner, Underhill, and Williams.

Reading of the minutes was dispensed with. First order of business was the appointment of delegates to the Veterinary Medical Association of New Jersey. The President appointed Drs. Pearson, Marshall, and Hoskins to represent the Keystone Association at Trenton, January, 1903.

Dr. C. J. Marshall, who was to report upon "Influenza," asked to be given until February meeting, when he would be prepared to report on the same.

The President announced the subject for discussion was "Lameness of the Hind Extremities." Dr. Hoskins was called upon to open the subject, in which he discussed the difficulty in making a differential diagnosis of hock lameness and lameness of the lower portion of the extremity, especially where the foot was not rested on the ground, reciting a number of cases of obscure hock and pastern lameness.

Dr. Charles Lintz reported upon several similar cases of the hock-joint. Dr. Charles Williams suggested the more frequent use of cocaine for diagnostic purposes. Dr. Kooker reported an obscure case of hock lameness, where swelling, heat, tenderness, and extreme lameness presented themselves. This case afterward developed a suppurative process at the base and hollow of the heel, which terminated in a deep slough almost to the navicular articulation. Dr. Thomas B. Rayner discussed nail wounds of the hind feet. Dr. Charles J. Marshall brought up the question of our right to remove rubber pads in examination for soundness. Dr. W. W. Drake reported very great benefit derived from the use of pads in hock lameness in the hind extremities and where there was a marked disposition to wear out the toe of the shoe with but little wearing of the calkings or wings of the shoe. The subject was further discussed by Drs. Mecray and Conard, after which the discussion closed.

The President then asked Dr. Hoskins for some remarks relative to the outbreak of foot-and-mouth disease in the New England States. After referring to the long period of time that elapsed before active measures were taken for the suppression of the disease, he specially referred to the excellent services rendered by the State Veterinarian of Pennsylvania in protecting the State's interests from the danger of an unjust quarantine proclamation by Governor Yates, of Illinois. The active measures adopted by the State Live-stock Sanitary Board in preventing our great foreign commercial interests from suffering from such unwise and uncalled-for measures, was very favorably commented upon by Drs. Marshall, Conard, and the President, and on motion a committee of three was appointed, consisting of Drs. Marshall, Underhill and Hoskins, to prepare resolutions commendatory of the prompt and resolute action of our State Live-stock Sanitary Board and our State Veterinarian, after which, on motion, the meeting adjourned.

W. Horace Hoskins,
Acting Secretary.

---

### SCHUYLKILL VALLEY VETERINARY ASSOCIATION.

The semi-annual meeting of this Association was held on Wednesday, December 17, 1902, at the Board of Trade Room, Reading, Pa., with Dr. Otto G. Noack, President, in the chair, and Dr. W. G. Huyett acting as Secretary. On roll-call the following members answered to their names: Drs. D. R. Kohler, Boyertown; G. A. Wehr, Denver; Otto G. Noack, Reading; F. H. McCarthy, Pottsville; W. G. Huyett, Wernersville; F. H. Schneider,

Philadelphia; S. G. Burkholder, Rothville; I. C. Newhard, Ashland; E. D. Longacre, Shenandoah, and W. S. Longacre, Mantz. Among the visitors were Drs. Leonard Pearson, University of Pennsylvania; E. M. Ranck, Glenolden; Jacob B. Leber, Ephrata, and Jacob N. Becker, Palmyra.

The minutes of the previous meeting were read and approved. The Treasurer, Dr. F. H. McCarthy, handed in a satisfactory report, showing a balance of $35 in the treasury after all bills had been paid. The President delivered his address, which was concise, as usual, but very comprehensive. After dwelling upon the work this Association has accomplished, he referred to the duties of the veterinarian in relation to the agricultural interests, especially in connection with the spread of infectious diseases communicable to man. He furthermore expressed a strong desire for a higher standard of the profession, claiming that a great advance to that effect would be gained by having all veterinary colleges under State supervision, instead of being private institutions.

The Association received its charter, which was read by the Secretary. A motion was made and carried that the same be accepted as satisfactory, thus rendering the organization an incorporated body. A few favorable remarks were given by Drs. Wehr and Kohler, delegates to the State meeting. The Society then adjourned for luncheon, convening again at 1.30 P.M.

The President offered the following resolutions:

WHEREAS, By the change of Governor the question of appointment of State Veterinarian arises; and

WHEREAS, The present incumbent, Dr. Leonard Pearson, has shown himself able and fully competent in the execution of the duties required by this office; be it

*Resolved*, That this Association recommends and urges his reappointment for this office to Governor-elect Pennypacker.

WHEREAS, A bill known as the Anti-vivisection Bill is introduced in the United States Senate to prohibit and make unlawful experiments on living animals; and

WHEREAS, The passage of such act would seriously interfere with the study of medical students; therefore be it

*Resolved*, That this Association protests against the passage of said act, and shall urge the Senators of this State to try and defeat this bill.

WHEREAS, This meeting of the Schuylkill Valley Veterinary Association has been one of the most successful that has ever been held by this organization; and

WHEREAS, This was only possible through the courtesy and kindness of Drs. Pearson, Ranck, and Burkholder to attend and favor us with their instructive essays; be it

*Resolved*, That the thanks of this Association are hereby extended to them for their aid in making this our meeting a success, and we thereby express our deep appreciation of the same.

These resolutions were approved as read and ordered spread upon the minutes. A recess was then given for the collection of dues. After adjournment some controversy followed in regard to a letter received by the Secretary from Dr. William Moyer, who, from the tone of the letter, seemed to be offended by a letter addressed to him, notifying him of his delinquency to the Association. The question being duly considered at the meeting, the Secretary was advised, after a motion had been made by Dr. Newhard and seconded by Dr. McCarthy to that effect, to write him an encouraging letter to pay his arrears promptly and become a member in good standing.

The Chairman of the Committee on Intelligence and Education was called upon, and Dr. W. S. Longacre responded with a few appropriate remarks.

Dr. D. R. Kohler, Boyertown, read a paper on the subject of "Parturient Paralysis." It was very practical, afforded some instructive points, and was thoroughly discussed by most of the members and visitors present. A motion was made and carried, tendering a vote of thanks to Dr. Kohler for his valuable paper.

Owing to Dr. Wehr not being fully prepared with his paper upon "Pleuro-pneumonia," he favored the audience with a general oral talk upon the subject, which was highly appreciated. The subject being very familiar, and a disease commonly met by every practitioner, much was commented upon the treatment. Dr. Pearson narrated the latest treatment followed at the University. He prefers strophanthus to digitalis for weakness of the heart's action, owing to the latter drug producing an intermittent pulse after using for three days. He also considers it advisable to draw off the serum as soon as clear symptoms are in evidence, as he states the operation is too often performed with results fatal, in which case success might prevail if performed earlier. The subject was also discussed by Drs. Wehr, Longacre, E. D. Kohler, Huyett, Newhard, and Noack.

Dr. E. M. Ranck, Glenolden, favored the Association by an ably prepared paper on "Blood Sera," after which he was called upon by the President to give a general talk upon vaccines and antitoxins, he being an authority of no mean repute along that line. He said, in part, in the production of antitoxins, the selection of the horse is a matter of extreme importance, this work being under the immediate personal control of skilled veterinarians, who fix an absolute and invariable standard as to the char acter of the animal employed, each horse having to withstand a rigid physical examination, after which he is injected with tuberculin and mallein. After this inspection, the selected horses are placed and kept in a specially constructed stable that is a model of sanitary perfection. A very small proportion of horses meeting the rigid requirements of physical soundness and health will yield antitoxin of sufficient unit strength, and in consequence those are discarded.

The toxins are elaborated from the specific germ of the disease, cultivated in some suitable culture media. After the expiration of the period of time required to obtain the greatest strength, the bacteria are killed by the addition of trikresol to the cultures, which are then repeatedly filtered in order to remove the destroyed germs.

The definite unit strength of these toxins is determined by a series of injections into guinea-pigs, thus ascertaining the minimum quantity of toxin which proves fatal to a guinea-pig within a certain period of time. This quantity of toxin (unit strength) is now injected into the horse under aseptic conditions. These injections, small at first, are repeated at regular intervals in order to obtain the degree of tolerance of immunity a horse gradually acquires to the influence of the toxins upon the system, and the blood develops the property of neutralizing these toxins. The injections of toxins are repeated in gradually increasing doses once every week until the horse receives and tolerates, without untoward symptoms, a quantity of toxins equal to one thousand times the amount of the first injection, this process consuming, on the average, from four to six months.

The horse is now bled into a previously-sterilized parchment-covered jar, which is then allowed to stand for a few days, when the serum collects on the top, and the clot or solid constituents settle to the bottom of the jar. The serum is now removed by a tube and transferred to sterile jars. Contamination of the serum, he says, is absolutely impossible, as at no stage of the process of antitoxin preparation is it exposed to the outside air.

The antiseptic trikresol is now added as a preservative, after which the strength of the same is determined by experimenting upon guinea-pigs.

The subject was discussed by Drs. Schneider, Wehr, Newhard, and others. A vote of thanks was extended to Dr. Ranck for his instructive remarks.

Dr. Leonard Pearson entertained the audience for some time with a paper entitled "The Veterinary Teacher and Practitioner," after which he described his trip to the New England States in order to investigate the outbreak of the foot-and-mouth disease in that section. He gave an excellent general report, which was highly appreciated. He also referred to the experiments upon the immunization of cattle against tuberculosis, conducted at present by Dr. S. H. Gilliland, Assistant Bacteriologist of the State Live-stock Sanitary Board, and himself. Some experiments had been conducted by McFadyean in June, 1901, and in March, 1902, also by Von Behring in December, 1901, but the observation thus ascertained from their reports certify that their experiments are yet incomplete. Dr. Pearson says that tuberculosis of cattle has been the subject of special and extensive study and experimentation in the laboratory and research station of the Pennsylvania State Live-stock Sanitary Board since 1896, but it is only of recent occurrence, after many experiments, that the results prove encouraging in the direction of immunization. Much enthusiasm was expressed by the audience upon the remarks of Dr. Pearson, as many of the members were unaware of such existing project, and all present were heartily in sympathy with such a stride of advancement in our profession. We all sincerely hope the experiments under way may prove successful, thus restraining the most insidious contagious disease upon the earth.

Dr. S. G. Burkholder's essay, see page 7, "An Historical Essay on the Relation of the Veterinarian to the Medical Profession," was replete with interesting and instructive thoughts.

A vote of thanks was extended Drs. Pearson and Burkholder, and resolutions passed to that effect.

At 4.30 P.M. a motion was made and seconded to adjourn.

W. G. HUYETT,
Corresponding Secretary.

---

### VETERINARY MEDICAL SOCIETY OF THE UNIVERSITY OF PENNSYLVANIA.

At the annual banquet of the Veterinary Medical Society of the University, given at the Bingham House, Tuesday evening, December 23, 1902, fifty members of the Society were in attendance. They entertained as their guests the Provost and Vice-Provost of the University, Dean Pearson and the members of the Veterinary Faculty. The room in which the dinner was served was tastefully decorated with flowers and with red and blue flags and bunting. Harry E. States, '03 Vet., the President of the Society, acted as toastmaster.

Among the invited guests and speakers were Dr. Charles C. Harrison, Dr. Edgar F. Smith, the Vice-Provost; Dr. Leonard Pearson, dean of the Department and also State Veterinarian, and the following members of the veterinary teaching staff: Dr. John W. Adams, Dr. M. E. Conard, Dr. S. H. Gilliland, Dr. W. Horace Hoskins, Dr. John Harshberger, and Dr. E. Stanton Muir.

The Provost was the first guest who was called upon to respond to a toast. He spoke very effectively on the importance of the live-stock industry and its bearings on those manufacturers who have to get their material from that source. He also dwelt upon the importance of veterinary science in maintaining the health of the live-stock and the prevention of disease, and concluded with a few touching remarks on his interest in the welfare of the Department. Vice-Provost Smith followed Dr. Harrison, speaking of the University generally. Dean Pearson spoke on the Veterinary Department as it is, its importance, and the outlook for its future. The Dean was followed by several different members of the Veterinary Faculty who made interesting remarks.

The Society is an undergraduate body and has been in existence almost since the founding of the Department at Pennsylvania. Its present membership consists of sixty-five students, recruited from all three classes in the Department. Harry E. States, '03, is President; Charles H. Woodruff, '03, Vice-President; John H. Morris, '04, Secretary, and W. D. Fuller, '03, Treasurer.

---

### ONTARIO VETERINARY MEDICAL ASSOCIATION.

The annual meeting of the Association was held in the Veterinary College, Toronto, on December 24, 1902.

The President, Dr. J. H. Tennent, was in the chair. He opened the meeting by an excellent address, in which the object in view was evidently the mutual improvement of members in all branches of veterinary science, both practically and theoretically, and the advancement of the position and interests of the veterinary profession in the Province of Ontario. Several of the points brought up elicited considerable but amiable discussion, in which many members participated.

The minutes of the previous meeting were read and approved.

The Secretary-Treasurer and Registrar's report was then presented. It showed a large amount of correspondence, considerable printing, and a new copy of the *Register* issued, containing a full list of those registered, in accordance with the act of incorporation of the Ontario Veterinary Medical Association, complete up to July 31, 1902; also containing the Act of Incorporation.

Fifty-five graduates have registered since the last annual meeting. The finances are in good condition, there being $22 more in the hands of the Treasurer than at the opening of the meeting last year.

The following names were proposed and duly elected to membership: R. H. Cook, V. S., Malton; J. M. Young, V. S., Oil Springs; J. D. McLeod, V. S., Harrison, and A. E. James, V. S., Ottawa.

The business routine being concluded, at the invitation of Prof. A. Smith, the members adjourned for lunch.

The meeting opened again immediately after lunch.

Dr. J. D. O'Neil read an excellent and practical paper on "Soundness and Unsoundness in Horses, and the Duties and Liabilities of the Veterinary Practitioner in Successfully Acquitting Himself in the Performance of this Necessary and Important Duty."

Dr. S. E. Boulter contributed an article on a case of tetanus in the horse —evidently a very severe case.

Dr. R. Barnes also contributed a paper on "Tetanus" that was a severe case. The medicinal treatment consisted principally of frequent hypodermic injections of solutions of carbolic acid and glycerin in water. Complete recovery resulted after about forty days.

The reading of these papers elicited considerable discussion, in which a number of members participated. The discussions were instructive.

The President suggested that some of the members should volunteer to attend the next annual meeting and perform some of the interesting surgical operations before the members for mutual benefit.

In response, Dr. George would operate, and Dr. Brenton, of Detroit, was suggested as the surgeon to perform an operation for "roaring."

Dr. Stevens exhibited a very ingenious instrument of his own for grasping the fœtus in cases of difficult parturition.

Dr. Rutherford, of Ottawa, Chief Veterinary Surgeon of the Dominion of Canada, gave an eloquent and stirring address. He had resided and practised in the Province of Manitoba for several years. He said that no man can legally practice veterinary science in that Province unless he is a member of the Manitoba Veterinary Medical Association, and he trusted that the members of the Ontario Veterinary Medical Association by standing shoulder to shoulder would eventually obtain the same result in Ontario. He gave a most interesting account of the meeting of the American Veterinary Medical Association, recently held in Minneapolis, of which he had been elected Vice-President. He had attended that meeting. He said that the clinical work performed there was done by the most skilful practitioners in America. He had succeeded in inducing that Association to hold its next meeting in the city of Ottawa, Canada, and he strongly urged the members of the Ontario Veterinary Medical Association to attend that meeting, which will be held in September next.

The President and Dr. D. K. Smith were appointed to represent this Association at the American Veterinary Medical Association meeting in Ottawa, also to aid Dr. Rutherford in entertaining its members there, and the sum of $100 was appropriated to be forwarded to Dr. Rutherford to pay a share of the expenses of the American Veterinary Medical Association in Ottawa.

Among the most important communications read was one from the President of the Industrial Exhibition Association of Toronto, requesting the cordial approval and active support of the Ontario Veterinary Medical Association in holding a Dominion Exhibition in Toronto in 1903. A resolution to that effect was passed at once without a dissenting voice.

There having been some discussion on breaches of professional ethics, the Committee on the Revision of the By-laws requested permission to defer their report until the next meeting.

The election of officers then took place, with the result that all the officers of the Association were re-elected.

The sum of $25 was voted for a medal to be presented for competition to the graduating class of the Ontario Veterinary College at the next spring examinations.

Adjourned. C. H. SWEETAPPLE, V.S.,
Secretary.

---

### WOLVERINE VETERINARY MEDICAL ASSOCIATION.

THE second annual meeting of the Association was called to order in the Council room of the city of Lansing, at 2 P.M., by the Vice-President. President, Dr. W. A. McLean, being detained at home by sickness in his family, sent the following address, which was read by the Secretary:

GENTLEMEN OF THE WOLVERINE VETERINARY MEDICAL ASSOCIATION: I regret very much that it is impossible for me to be present at our annual meeting of 1902, hoping at the same time that there will be the usual large attendance, and that you will profit by each other's experience and research; also, that the different papers will be thoroughly discussed. Shall be much pleased to hear a favorable report from the Committee on Legislation, and for the members of the Association to combine their efforts and work for the passage of a bill to protect the interests of the veterinarians of the State of Michigan. Vice-President Hesseltine will be in a position to help us to some extent in this matter. The passage of such a law has been hanging fire for a number of years, and I don't see that we are any nearer proper veterinary legislation than many years ago. It is absolutely necessary that we should frame a law so as to help elevate the profession in this State.

I gave you my ideas at our last semi-annual meeting, held at Flint last July, which can be found in the minutes, and no doubt can be improved. I am anxious to see the time come when every person registered in this State shall be a graduate of a three years' college. We can by legislation *compel* the Ontario Veterinary College and the Grand Rapids Veterinary College to go to a three years' course; by so doing we will be doing justice to ourselves and to the citizens of the State.

I have written Dr. Palmer, of the Michigan State Veterinary Board, in regard to this matter, but as yet have no reply, although some six weeks have elapsed.

I am of the opinion that we have selected the wrong time in the year for our annual meeting—during the Christmas holidays—when many wish to take advantage of the excursion rates to visit friends at a distance; therefore would suggest a change of date of our annual meeting.

I am also under the impression that two meetings a year are too many, one of my reasons being based on the expense for persons living at a distance. I think one good meeting would be much better.

I am not a candidate for re-election for President.

Hoping you all may enjoy a prosperous and happy New Year,

I am as ever, yours,

W. A. MCLEAN, V.S.,
President.

The Vice-President, Dr. Hesseltine, then read the following address:

GENTLEMEN: Again I am given the pleasure and privilege of the opportunity of being with you at this the second annual meeting of the Wolverine Veterinary Medical Association.

As Vice-President of this Association and Chairman of our Disease and Legislative Committees, I realize that I have certain duties to perform at these meetings, but this time I must ask that I be excused in behalf of my brother members from making a full report and in covering the field thoroughly upon these subjects, of which jurisdiction I am held responsible by reason of my position in connection with this Association and my profession. I wish to say further that I would not ask for any excuse to be accepted.

For my part, in behalf of the members of this Association, if it were not for the fact that since our last meeting, in which I took a great interest in seeing that all were entertained; that the meeting in general was a success; that my time has been constantly occupied along other lines, yet those lines have not been of a detrimental character in accordance with the position I am holding in relation to my profession and my Association.

The various editorials that have appeared in our daily papers no doubt have informed many of you the position I have taken and the untiring efforts that I have made in trying to develop and incorporate a universally recognized scientific system for the teaching of the American horseshoers the true fundamental principles of a comparative branch of veterinary medicine and surgery, to be known as comparative digitalotomy. And while here I wish to say in behalf of my brother horseshoers of America that they wish to invite a more kindly feeling between the veterinarians and the masters, and that they do most heartily extend to the veterinarians fruits of fraternal unity in an effort to remove the present misunderstanding, grievances, and indifferences which to-day exist throughout this great united kingdom.

At the annual meeting of the M. M. H. A., held at Grand Rapids, September 1st and 2d, the subject in relation to the masters keeping cabinets of patent medicines in their shops for sale was brought up for discussion by myself. The subject received far more consideration at the hands of the delegates than I anticipated, and should I at this time attempt to predict the outcome of such a practice I would feel safe in saying that I fully believe that the day is not far distant when such practice will be completely abolished by the master shoers of America, and most any other practice that would tend to conflict or interfere in any way with the practice of the veterinarian would likewise be done away with; and in recognizing such movements on the part of the American shoers I feel that it behooves us as veterinarians to take such step as will show a return of appreciation upon our part as professional men.

American horseshoers fully realize to-day that they are no longer representatives of a trade, but a profession. They are in every sense of the word professional men; they have taken steps that will lay the future foundation for the true teaching of a comparative branch of science which will be known as digitalotomy. . . . The subject of legislation is very important and requires careful thought; true legislation will protect the practice of veterinary medicine and surgery. I have given the subject a great deal of thought and study, coming to the following conclusion:

That we can never expect the fulfilment of our desires and aims while divided among ourselves. The first steps that we should take are such as will cause complete unity of our ranks; to place ourselves in harmony, one with the other, using every effort to consolidate the two associations in one, because, never until we are united can we grow and prosper. . . . We need legislation to protect and govern the veterinarian; we need legislation to protect domesticated animals from the many contagious diseases to which they are heir; we need legislation to protect our meat food supply; lastly, but not least, we should have proper legislation to protect the veterinarian in collecting a proper compensation for services rendered and medicine furnished for the treatment of diseased animals. . . . If we were a united body we could demand our rights of the Legislature; but so long as we are divided, we cannot expect representatives of the House or Senate to heed our wants, needs, or desires.

Both sides of our divided forces have friends in the Legislature, therefore we must expect defeat. It is not money that we need to obtain legislation, but friendly relations among ourselves.

In addition to the foregoing I would suggest that our worthy President appoint a committee to draft a set of resolutions that may be acted upon by this Association, and a copy of it be sent to the Secretary of the Michigan Veterinary Medical Association, praying for unison and harmony and consolidation.

Minutes of the last meeting were read and adopted.

Report of the death of Dr. B. O. Johnson was officially announced, and a committee was appointed by the Chair to draft resolutions. The following named gentlemen were appointed: Drs. McCall, Elliott and Winegar.

Hon. Charles Staley, President of the Michigun Horseshoers' Association, by invitation, gave a very interesting address on the subject of "Higher Education of Horseshoers," and incidentally touched upon the subject of discord now existing in the veterinary profession of this State, suggesting that a remedy be sought, giving as his opinion that nothing short of amalgamation of the two associations could ever bring about unison in our ranks, and that he thought proper steps taken in this direction would be fruitful; that he had travelled over a goodly portion of the State and felt confident that he knew whereof he was speaking.

The remarks of President Staley were received with applause, and as he closed his remarks Dr. Conkey arose and gave to him a brief history of circumstances which culminated in the birth of the Wolverine Veterinary Association, the principal reason being the fact that the old Association refused to admit graduates of the Grand Rapids Veterinary College, which was a violation of the law under which they are chartered. The law plainly states that "any veterinary surgeon who has complied with the laws regulating the practice of the profession in this State may become members." However, if it is the pleasure of the old Association to amalgamate, we say amen to it, and further will vouch for each and every member of this Association doing likewise. In the meantime we are in a good healthy condition as to membership, numbering forty-one in good standing, with the prospect of a material increase, and we firmly believe that within two years we shall outnumber the old Association; but harmony is what we want, and I for one am ready to sacrifice much if we can only have harmony.

Dr. P. Hesseltine was the next speaker, and dwelt long and loud upon the hope of unison and harmony in our ranks; he also strongly advocated reciprocal relation between the Veterinary Association and the Master Horseshoers' Association. Occupying the chair, the doctor appointed a committee to draft resolutions along these lines.

A recess was taken to allow the committee time to draft resolutions on the death of our ex-President, Dr. B. O. Johnson, and also regarding the horseshoers and the Michigan State Veterinary Associations.

Meeting again called to order, and the following resolutions were read and ordered spread upon the minutes of the Association, and a copy to be sent to his widow at Benton Harbor, Mich.:

WHEREAS, Divine Providence has seen fit to remove from the midst of this Association our beloved brother and ex-President, Dr. B. O. Johnson, of Benton Harbor, Michigan, on the twenty-ninth day of November, 1902.

*Resolved*, That the members of this, the Wolverine Veterinary Medical Association, do extend heartfelt sympathy to his widow and children in their loss and bereavement of a kind and loving husband and father, assuring them that we feel deeply the loss of his presence and silence of his voice at our meetings.

*Resolved*, That these resolutions be spread upon the minutes of this meeting and that a copy of them be sent to the family at Benton Harbor, Michigan.

DR. A. E. MCCALL,
DR. WM. ELLIOTT,
DR. AMOS WINEGAR,
Committee.

The following resolutions were then adopted:

*Resolved*, That this Association, by a unanimous vote of all members present, extends the right hand of good fellowship to the Michigan Veterinary Medical Association; that in the consideration of the existing conditions which, up to the present time, have prevented the growth, advancement, and protection to the veterinary profession of the State of Michigan, do hereby ask that this subject be duly considered by a committee to be appointed at the first session of the annual meeting of the Michigan State Veterinary Association, for the purpose of effecting a complete settlement of all disputes, grievances, and differences now existing among the leading representatives of the two associations, said committee to be clothed with power to act.

*Resolved*, That the incoming Executive Board of this Association be instructed to meet and confer with said committee of the Michigan State Veterinary Medical Association, both committees having full power to act, so that the findings of said committees shall be final and binding upon their respective associations.

We hereby attach to and make a part of these resolutions our sentiment, position, and expression:

*Resolved*, That the Wolverine Veterinary Medical Association does extend the right hand of fellowship to the Michigan Horseshoers' Association; and also, be it extended to the National Horseshoers' Association of the United States of America.

*Resolved*, That the members of this Association use all reasonable means and their best effort for the promotion of harmony between the horseshoers and Veterinarians of the United States.

The Committee on Legislation having failed to offer anything for the consideration of the Association, the bill which appeared in the November number of the *National Veterinary Review* was placed in the hands of a special committee, and after some remodelling and a great deal of argument pro and con the following was unanimously adopted :

*Resolved*, That the following veterinary bill, which has been prepared by a special committee of this Association, and has been discussed in open meeting and found to be acceptable to the good wishes of the members of this Association, embodying all the aims and desires for the betterment of the present conditions, for the protection of the stock grower and practitioner of the State of Michigan.

*Resolved*, That this bill be considered with the foregoing resolutions and be brought before the Michigan Veterinary Association while in session, and that it be requested that a report of the same be submitted to the committee of the Wolverine Veterinary Medical Association.

Respectfully submitted,

DR. W. N. ARMSTRONG,
DR. CHAS. DONALD,
DR. L. L. CONKEY,
Special Committee.

Adopted unanimously.

A BILL

To regulate the practice of the veterinary profession in all of its branches in the State of Michigan, and to classify all those who are now engaged in the practice of veterinary medicine and surgery in any of its branches in this State.

*The people of the State of Michigan enact:*

SECTION 1. That all persons now engaged in the practice of veterinary medicine and surgery in this State, and holding a diploma issued by a recognized veterinary college, shall be known as the regular titled veterinarians of Michigan, and shall use the title or degree conferred by the college issuing their diploma; and if wishing to continue in practice shall, on or before July 1, 1903, file with the county clerk of the county in which they reside an application for registration.

All veterinarians registering under this section shall be eligible to any State appointment, and shall be exempt from jury duty in this State; their evidence shall be accepted when called upon in the capacity of an expert witness in all matters pertaining to the veterinary profession.

SEC. 2. All persons that have been registered by the Michigan State Veterinary Board by reason of having passed the examination of said Board, and all who may be registered in a like manner prior to July 1, 1903, shall be recognized as veterinarians of this State. The evidence of all such may be accepted as expert in any court of justice if called for that purpose.

SEC. 3. All men who have been in exclusive practice of the veterinary profession as a means of livelihood for five years or longer next prior to the passage of this act, and wishing to continue said veterinary practice, shall at any time prior to July 1, 1903, file with the county clerk of the county in which they reside an application for registration; said application bearing the signature of five freeholders of said county, sworn before a justice or notary public, and setting forth the fact that the applicant is personally known to them, and that he is qualified to practice under this act.

All those registering under this section shall be known as the Time Clause Veterinarians of Michigan, with the title of "T. C. V."

SEC. 4. Any person practicing the veterinary profession in this State and wishing to advertise his business in any manner whatsoever shall confine his title, degree, or abbreviations thereof to the section under which he is registered, viz., sections 1, 2, or 3 of this act.

SEC. 5. After July 1, 1903, no person shall be allowed to practice veterinary medicine or surgery in any of their branches in the State of Michigan unless he has been registered under preceding acts or shall become registered under the expressed provisions of this act. After the above date any person wishing to begin the practice of veterinary medicine or surgery in any of their branches in this State shall, before doing so, apply to and receive from the county clerk of the county in which he resides, a certificate of registration. All certificates of registration must be framed and hung in a conspicuous place in the office of the person to whom it has been issued.

Veterinarians residing outside of Michigan, on the border lines, and qualified to register under this act may register with the clerk of the county nearest to which they reside, and practice in this State,

*Provided*, That the State or county in which they reside shall have equally stringent laws governing the practice of veterinary medicine and surgery as the laws of this State, and shall accord to the veterinary profession of this State the same privileges.

SEC. 6. On and after the passage of this act it shall be unlawful for the clerk of any county in the State of Michigan to enter the name of any person upon the books of his office as a veterinarian, veterinary physician, or veterinary surgeon unless the applicant for registration shall furnish to said county clerk satisfactory evidence of his identity and qualifications to become registered under the provisions of this act, as hereinbefore provided.

From and after July 1, 1903, it shall be unlawful for the clerk of any county to register any person under this act except upon the presentation of a diploma issued to the applicant by a regularly incorporated veterinary college; said college shall have a curriculum of at least three years of six months each.

The county clerk may demand and shall receive a fee of one ($1.00) dollar for recording, filing, and issuing each certificate of registration as contemplated in this act.

SEC. 7. Any person violating any of the provisions of this act shall be deemed guilty of a misdemeanor, and shall be proceeded against by the prosecuting attorney of the county in which the offence is committed, and upon conviction shall be fined not less than twenty-five nor more than one hundred dollars, or he may be confined in the county jail for not less than thirty nor more than ninety days, or he may be punished by both fine and imprisonment, at the discretion of the court.

SEC. 8. All acts or parts of acts controverting the provisions of this act are hereby repealed.

Paper entitled "The Foot of the Horse" was read by Dr. Conkey. Discussion led by Dr. McCall, followed by Drs. Ackerson, Armstrong, Elliott, Winegar, Donald, Nelson, Compton, and Hesseltine, was very interesting and brought out many instructive points.

The following-named gentlemen were admitted to membership: Drs. E. L. Krieger, J. D. Bell, Amos Winegar, J. Wardle and G. H. Du Boice.

The name of Mr. Francois, of Kalamazoo, that came before the Association at the last meeting, held at Flint, and was to be ratified conditionally, was stricken from our books, he not having complied with the conditions.

Report of the Committee on Publication was called for. Dr. Conkey, chairman of this committee, reported that the proceedings of the last meeting had gone to the press direct from the stenographer, and that the committee had never seen them until they appeared in the *International Veterinary Review*. This accounts for the many errors that appeared, to the discomfort of not a few of the members. Some corrections have been made, and the requisite number of copies are to be filed with the different State institutions by the Secretary this month.

On motion of Dr. Armstrong, seconded by Dr. Ackerson, the report was accepted.

The Secretary was instructed to send copies of the resolutions on the subject of the amalgamation of the two associations to Drs. Giffen, of Detroit; Jopling, of Owasso; H. Smith, of Albion; J. Black, of Richmond; H. F. Palmer, J. J. Joy, and S. Brenton, of Detroit; Charles Smith, Saginaw; Mr. Buckley, of Detroit; Mr. Staley, of Lansing, and Mr. Wm. Wheaton, of Grand Rapids, and a copy to each member of the Executive Committee of the W. V. M. A. The President and the Secretary of the National Horseshoers' Association is each to receive a copy.

Election of Officers. Dr. W. N. Armstrong, of Concord, was elected President; Dr. Donald, of Bay City, First Vice-President; Dr. McCall, of Memphis, Second Vice-President; and Dr. W. W. Thorburn, of Lansing, Secretary and Treasurer.

Banquet. At 7.30 an adjournment was taken, all repairing to Hotel Butler, where a sumptuous spread had been prepared, consisting of the most delicious fruits, nuts, and viands, both local and tropical, that money could procure. Seven courses were served in a most elegant and approved style. All were loud in their praise of the repast. A vote of thanks was tendered to Mr. I. M. Brown, proprietor of the hotel.

Dr. P. Hesseltine, of Flint, acted as toastmaster, and was responded to by Hon. Dr. Nottingham, a member of the House of Representatives; Hon. F. L. Dodge, an ex-member of the Legislature; Hon. Charles Staley, President of the Michigan Master Horseshoers' Association; Hon. Mr. Petty, ex-Organizer of Horseshoers, and a large number of the members present.

Installation of Officers. After the banquet an evening session was held in the hotel parlors at which the installation of officers took place. Dr. A. E. McCall was elected to fill a vacancy in the Board of Directors, to succeed Dr. E. E. Patterson, whose term of office had expired.

President Armstrong appointed the following-named gentlemen to read papers at our next annual meeting: Drs. Ackerson, McCall, Compton, Winegar, Donald, Elliott, Hesseltine and Conkey.

Adjourned to meet at Dr. Thorburn's hospital at 9 A. M.

The Association met at Dr. Thorburn's hospital at 9 A.M., January 1, 1903. There were an even dozen clinical subjects in the hospital awaiting the surgeon's knife. Four sows were castrated, two bitches, one male dog, two cats, and a number of cockerels. There were a few horses for examination. The work was quite well divided among the older members of the profession. This work consumed the forenoon.

After dinner the Association was called to order in the parlors of Hotel Butler at 1.30.

The President, Dr. W. N. Armstrong, appointed the following committees:

*Committee on Diseases.*—Drs. Nichols, Ackerson, Stevenson, and Compton.

*Committee on Finance.*—Drs. Nelson, Wardle, Dr. Winegar, and Elliott.

*Committee on Publication.*—Drs. Conkey, McCall, Bell, and Powers.

*Committee on Legislation.*—Drs. Thorburn, Donald, Hesseltine, and Patterson.

From 2.30 to 4 P.M. much routine business was transacted, after which the President declared all business complete, and opened an "Experience Meeting," which was heartily enjoyed by all present, every man having something to offer for the good of the profession.

At six o'clock a final adjournment was taken, thus ending the most pleasant, enjoyable, and instructive meetings in the history of the Wolverine Veterinary Medical Association.

L. L. CONKEY, V.S.,
Secretary *pro tem.*

